# Supplementary material for: Frequent, infinitesimal bottlenecks maximize the rate of microbial adaptation
Source: Genetics. 2023 Oct 7;225(4):iyad185. doi: 10.1093/genetics/iyad185 (PMC10697810; doi:10.1093/genetics/iyad185)
Supplement: iyad185_Supplementary_Data [file iyad185_supplementary_data.pdf]

# Frequent, infinitesimal bottlenecks maximize the rate of microbial adaptation: Supplementary Materials

Oscar Delaney, Andrew D. Letten, Jan Engelstädter

September 26, 2023

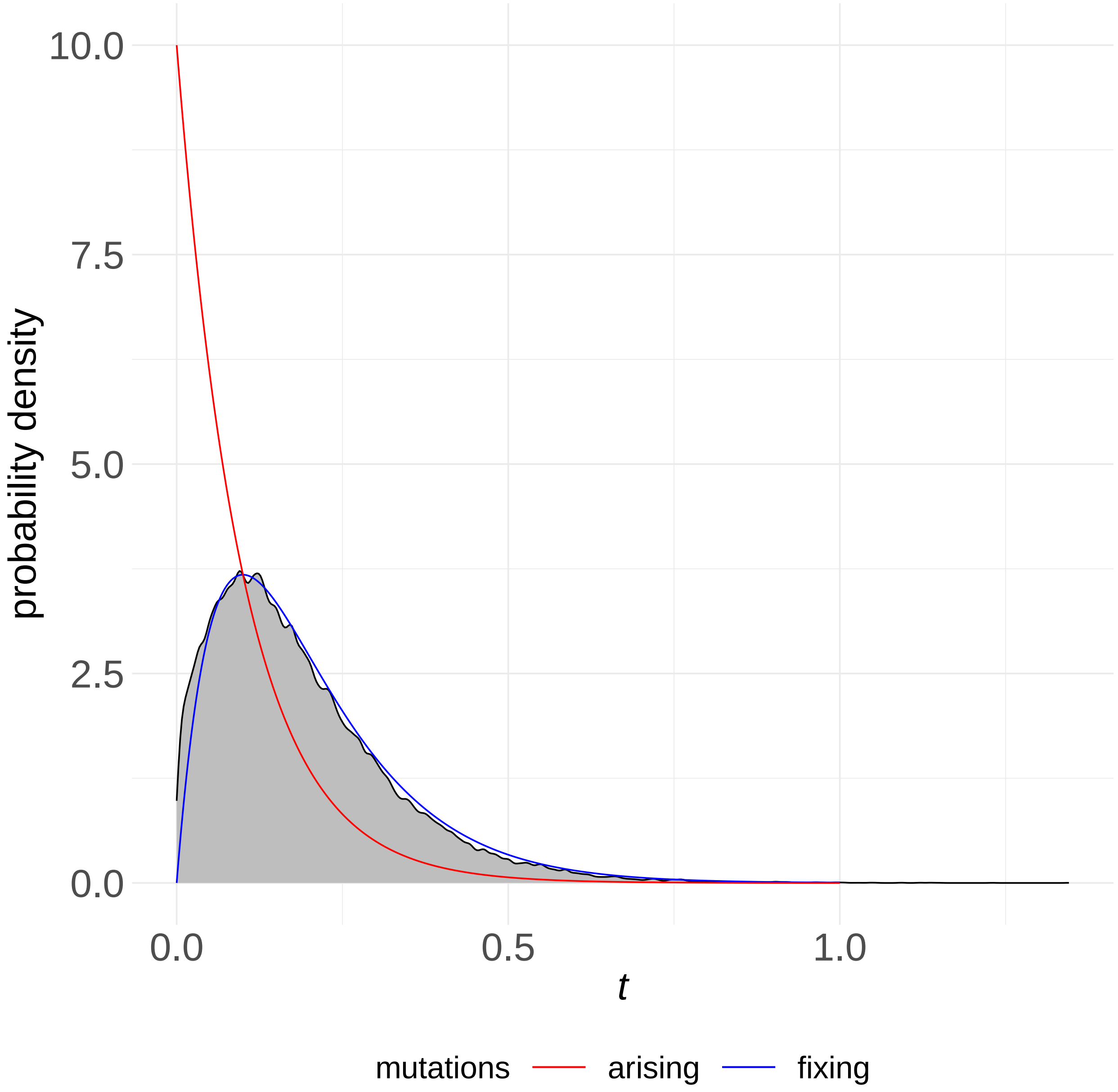


Figure S1: Distribution of selective benefits $s$ of beneficial mutations that fix. A kernel-density estimate was used to construct the distribution of $s$ for mutations that go onto fixation, which is compared against the theoretical distribution of $s$ for all mutations that arise, and just those that fix. The resource-unconstrained model was used, with parameter values: $N = 10^9, D = 10^{-0.1}, \mu = 10^{-8}, r = 1, \omega = 0.1, \tau = -\ln(D)$.

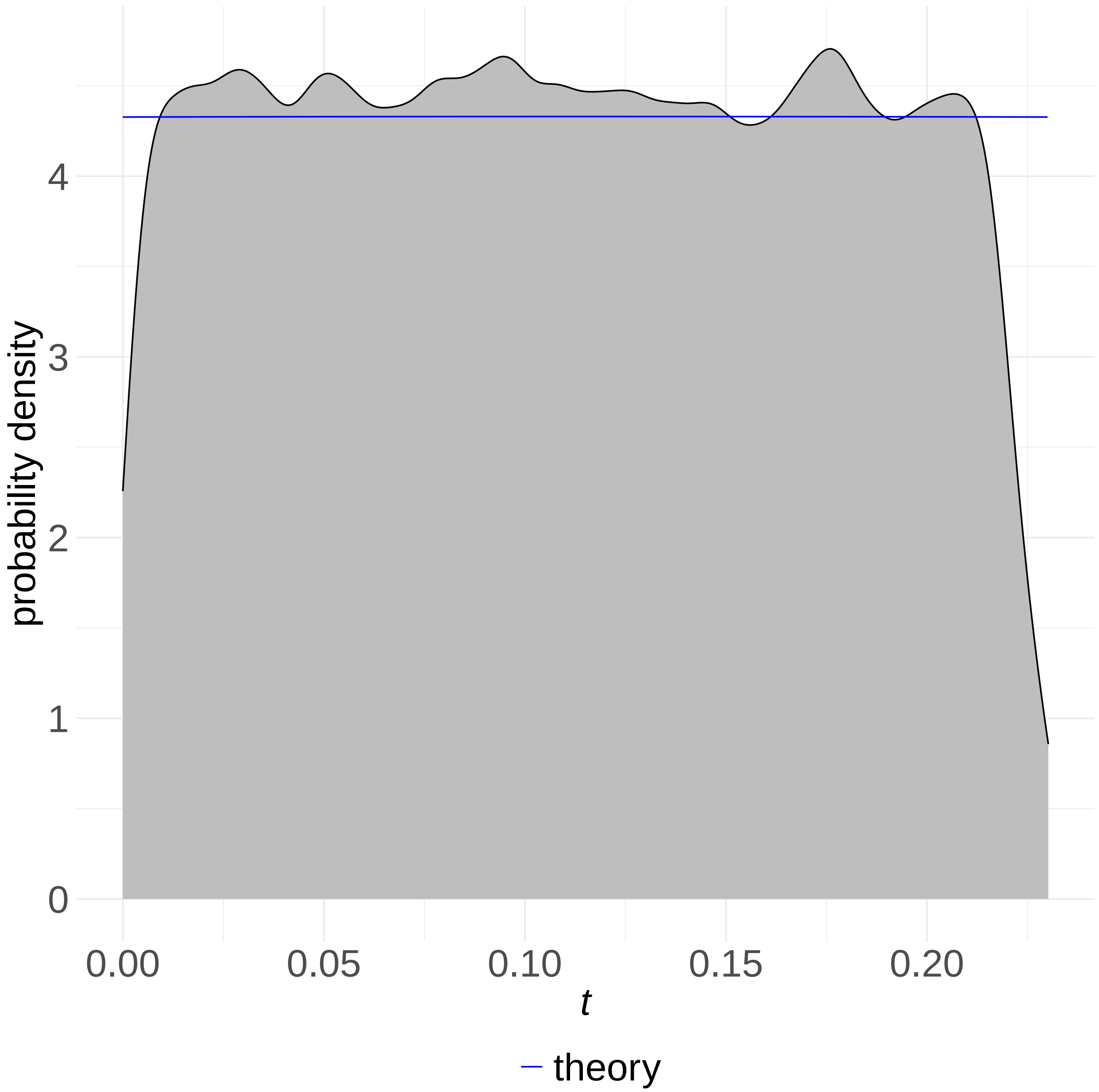


Figure S2: Distribution of times of occurrence $t$ of beneficial mutations that fix. A kernel-density estimate was used to construct the distribution of $t \mod \tau$ for mutations that go onto fixation, which is compared against the theoretical distribution, in this case indistinguishable from a uniform distribution. The resource-unconstrained model was used, with parameter values: $N = 10^9, D = 10^{-0.1}, \mu = 10^{-8}, r = 1, \omega = 0.1, \tau = -\ln(D)$.

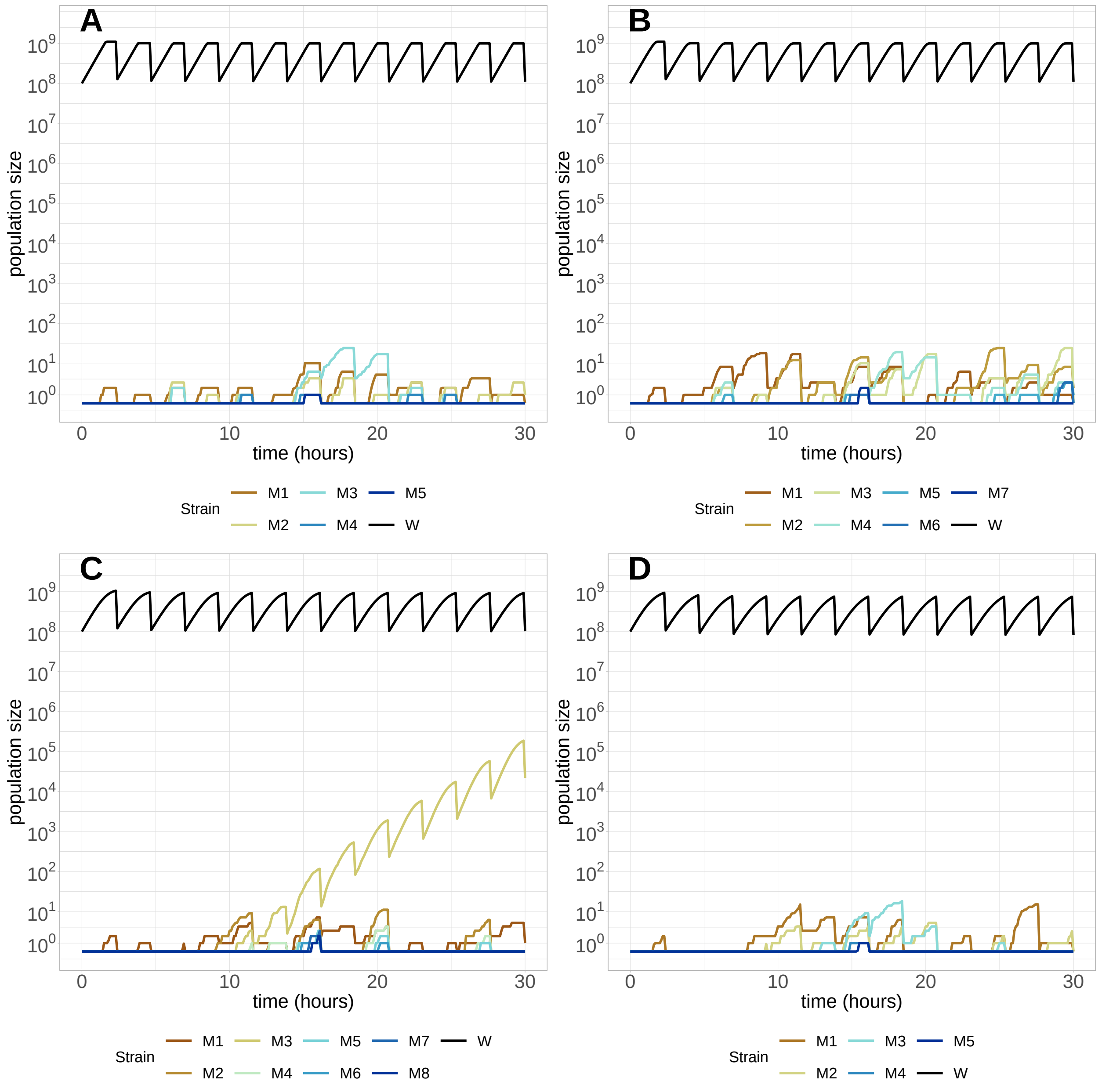


Figure S3: Example resource-constrained growth dynamics. Each panel shows one run of the simulation, with the resource-affinity parameter k ranging through $10^7$ (A), $10^8$ (B), $10^9$ (C), and $10^{10}$ (D). Other parameter values used were: $N = R_0 = 10^9, \mu = 3 \times 10^{-9}, r = 1.5\left(1 + \frac{k}{R_0}\right), \omega = 0.1, D = 0.1, \tau = -\ln(D)$. Values of $r$ vary with $k$ to ensure that the growth rate at $t = 0$ is constant for all $k$. This initial growth rate is higher than in the resource-unconstrained case so that the population does not go extinct when resource depletion leads to declining growth rates between bottlenecks. At the extremes, if $k \ll R_0$ the population grows almost exponentially until a relatively sudden halt when the resources are depleted, while if $k \gg R_0$ the Monod function is roughly linear and the populations grow logistically.

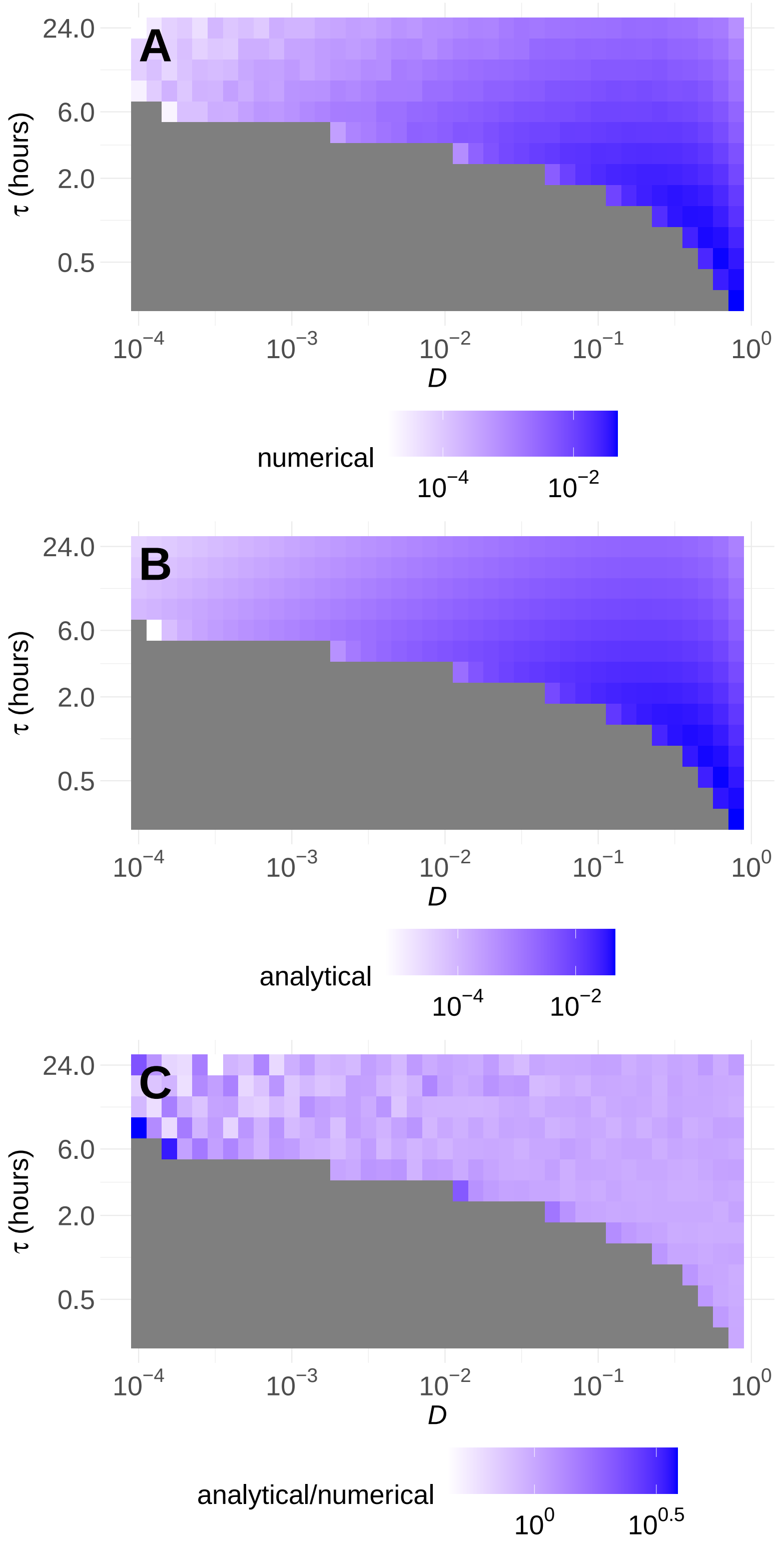


Figure S4: Adaptation rate in the $D-\tau$ landscape. Parameter values were: $N = R_0 = k = 10^9, \mu = 10^{-9}, r = 1.5\left(1 + \frac{k}{R_0}\right) = 3, \omega = 0.1$. In panel A each grid square represents 1000 simulation runs, while panel B shows the analytical solution as presented in the main text. Panel C shows the log ratio between the analytical and numerical results.

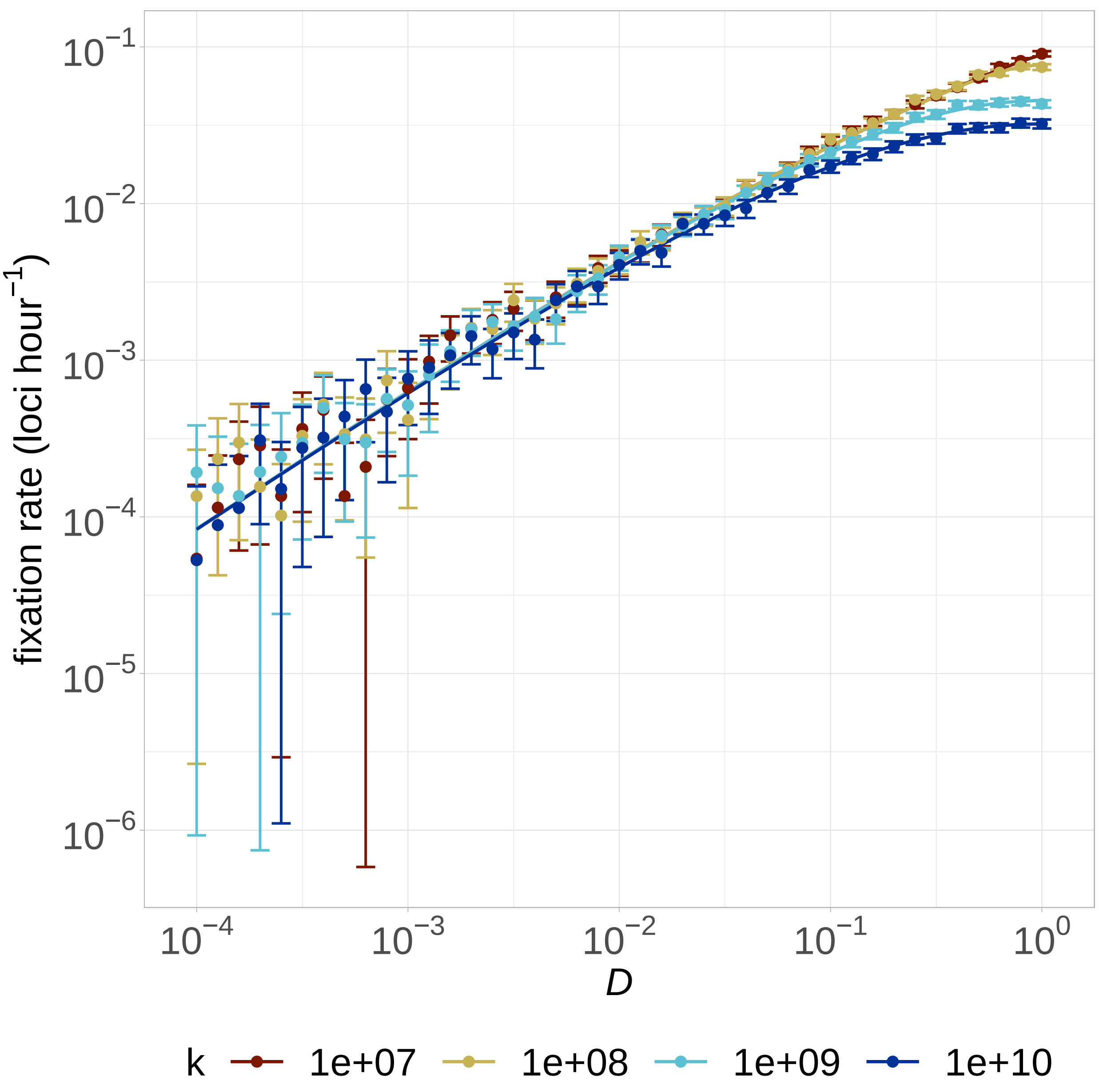


Figure S5: Optimal bottleneck size in resource-constrained growth. Each dot represents 1000 simulation runs for 20 hours each. The right-most data points at $D = 1$ were generated with the true chemostat model with a constant flow rate of 1 reaction volume per hour, with no bottlenecks. The beneficial mutation fixation rate was calculated for each run, and error bars were determined as the mean $\pm 1.96 \times$ se. This is compared with the theoretical approximation given in the main text. Parameter values were: $N = R_0 = 10^9, k \in \{10^7, 10^8, 10^9, 10^{10}\}, \mu = 10^{-9}, r = 1.5\left(1 + \frac{k}{R_0}\right), \omega = 0.1, \tau = -\ln(D)$.

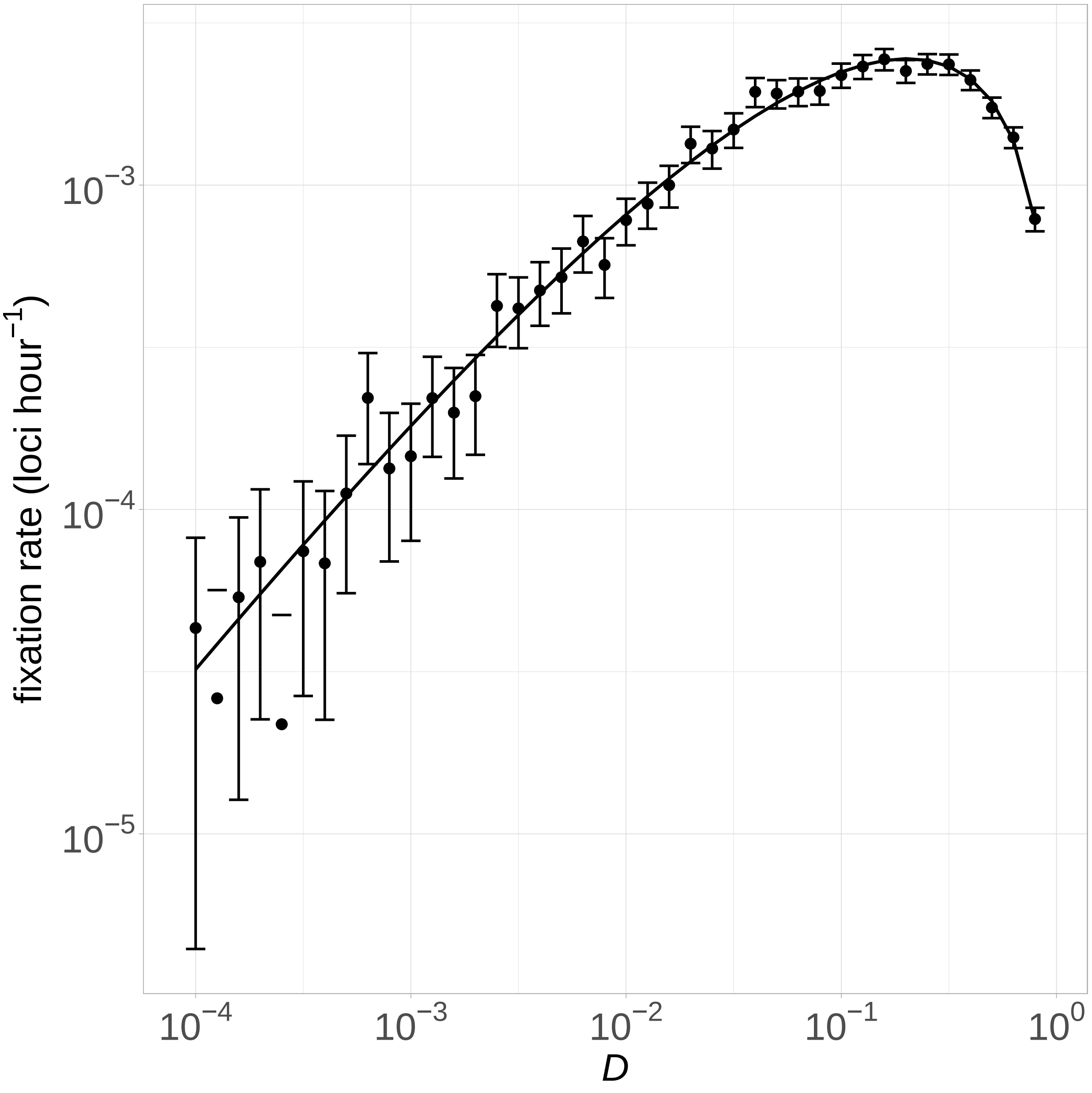


Figure S6: Adaptation rate when $\tau = 24$. Each point represents 2000 simulation runs for 50 hours each. The beneficial mutation fixation rate was calculated for each run, and error bars were calculated as the mean $\pm 1.96 \times$ se. The solid line shows the theoretical approximation given in the main text. Other parameter values used were $N = R_0 = k = 10^9, \mu = 10^{-9}, r = 1.5\left(1 + \frac{k}{R_0}\right) = 3, \omega = 0.1$.

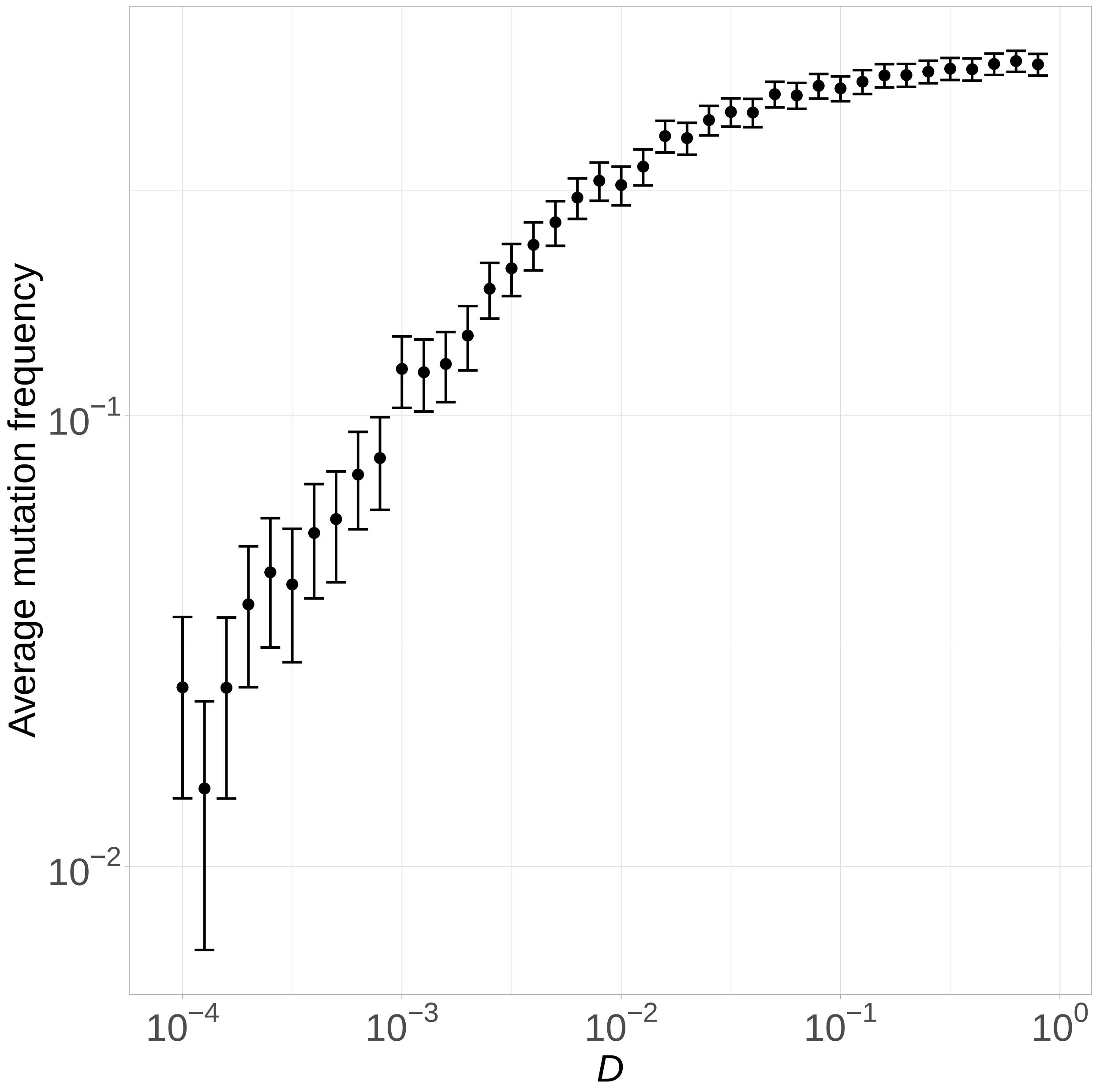


Figure S7: Adaptation under clonal interference. Each point represents 200 simulation runs for 1000 hours each using the genotype-based model with 4 mutable loci. The mean of the beneficial mutation frequency at each of the 4 loci after the 1000 hours across 200 replicates was found, and error bars were calculated as the mean $\pm 1.96 \times \text{se}$. The resource-unconstrained model does not work for longer runtimes as fitter strains replicate faster and go toward infinity. Therefore, the resource-constrained model was used, with parameter values: $N = R_0 = k = 10^9, \mu = 10^{-9}, r = 1.5\left(1 + \frac{k}{R_0}\right) = 3, \omega = 0.1, \tau = -\ln(D)$.

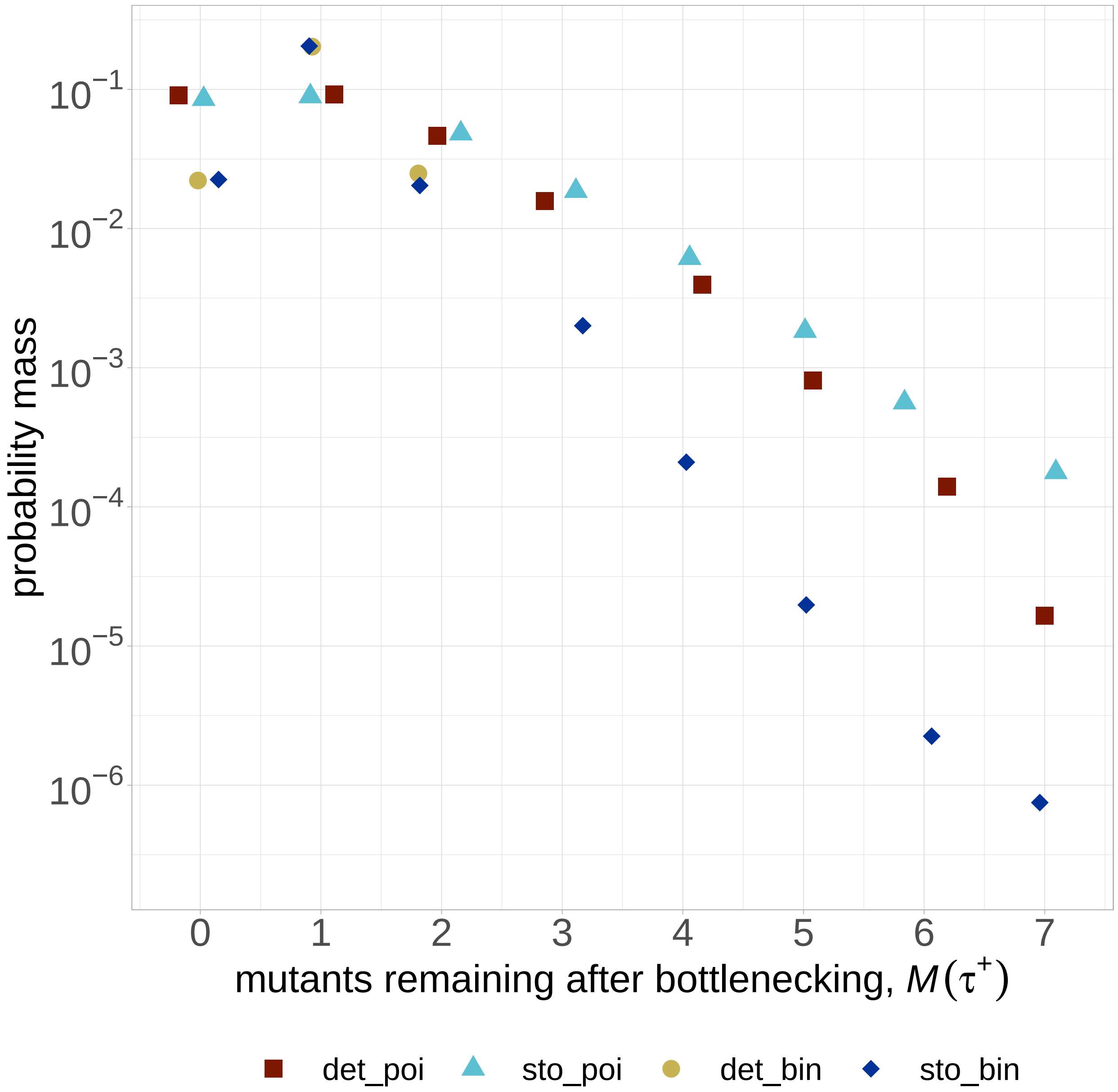


Figure S8: Probability mass function of the number of mutants surviving a bottleneck. Distributions were generated with $10^6$ random replicates of one mutant with selective benefit $s = 0.1$ growing for one resource-unconstrained growth period of length $\tau = -\ln(D)$ hours where $D = 0.9$ and $r = 1$, followed by a bottleneck. The four data series represent different possible methods, with the one used in this analysis being stochastic-binomial.
